# Knowledge domain and evolutionary trends of P2Y receptors in cardiovascular diseases: a bibliometric and altmetric analysis

**DOI:** 10.3389/fphar.2025.1731397

**Published:** 2026-01-20

**Authors:** Mengyu Yang, Xuanrui Chen, Shiyin Lie, Jingjing Li, Xuezhang Chen, Ju Chen, Zhiqiang Zhang, Liming Lu, Jingchun Zeng

**Affiliations:** 1 Medical College of Acu-Moxi and Rehabilitation, Guangzhou University of Chinese Medicine, Guangzhou, China; 2 Baoan Traditional Chinese Medicine Hospital, Shenzhen, China; 3 Foshan Hospital of Traditional Chinese Medicine, Foshan, China; 4 Zhongshan Hospital of Traditional Chinese Medicine, Zhongshan, China; 5 Department of Rehabilitation, The First Affiliated Hospital, Guangzhou University of Chinese Medicine, Guangzhou, China; 6 Guangdong Clinical Research Academy of Chinese Medicine, Guangzhou, China; 7 The First Affiliated Hospital of Guangzhou University of Chinese Medicine, Guangzhou, China

**Keywords:** altmetric analysis, bibliometric analysis, cardiovascular diseases, P2Y receptors, research

## Abstract

**Objective:**

This study employs a dual-metric framework integrating bibliometric and altmetric analyses to systematically map the knowledge domain and structural evolution of P2Y receptor research in cardiovascular diseases (CVDs) from 2005 to 2025.

**Methods:**

Data were systematically retrieved from the Web of Science Core Collection, Scopus, PMC, and Altmetric databases. By integrating network visualization (CiteSpace, VOSviewer), descriptive bibliometrics, and qualitative content analysis, this study mapped the structural evolution of the research landscape. Additionally, the correlation between scholarly impact and social visibility was quantified using Spearman analysis.

**Results:**

Our analysis encompassed 2,591 articles published between 2005 and 2025, from which the top 100 based on citation count and Altmetric Attention Score (AAS) were selected. The annual publication volume demonstrates sustained growth. The USA contributed 941 articles and holds a leading position, with scholars such as Dominick J. Angiolillo (132 articles) forming a core collaborative network focused on the clinical translation of P2Y_12_ antagonists. High-impact journals (Impact Factor >5) accounted for 80% of the publications, indicating a strong clinical orientation; the Journal of the American College of Cardiology received the most citations. The research focus has shifted from the antithrombotic mechanisms of early P2Y_12_ inhibitors like clopidogrel toward immune inflammation, myocardial regeneration, and precision medicine. Altmetric Attention Scores showed a high correlation with social media attention but only a moderate correlation with academic citations, whereas Mendeley readership correlated strongly with citations, revealing a divergence between social visibility and scholarly impact.

**Conclusion:**

P2Y receptor research has transformed from a singular antithrombotic focus into a multidimensional regulatory network. While P2Y_12_ antagonists remain clinically dominant, emerging frontiers focus on “de-escalation” strategies, immune-inflammation, and myocardial regeneration. Future progress relies on systematic evaluation of subtype selectivity and novel delivery systems, positioning P2Y receptor modulation as a promising avenue for precision cardiovascular medicine.

## 1 Introduction

Cardiovascular diseases (CVD_S_) are a major global public health threat (Ali et al., 2023; Tong et al., 2024), and their high morbidity and mortality have posed a serious challenge, affecting about 1.1 billion patients worldwide and more than 15 million deaths per year. With the aging of the global population and the transformation of lifestyle, it is a key issue to explore the pathological mechanism and develop effective prevention and control strategies (Gaidai et al., 2023; Lima et al., 2023; Wang X. et al., 2025).In molecular mechanism research, the P2Y receptor is the core target of platelet activity regulation, and its role in CVD_S_ development has become the focus of the field (Strassheim et al., 2020; Zhou et al., 2020; Sophocleous et al., 2025).

P2Y receptor belongs to the G protein-coupled receptor (GPCR) superfamily, which initiates downstream signaling pathway by recognizing extracellular nucleotides (such as ATP, ADP, etc.), and participates in key physiological processes such as vascular tone regulation, inflammatory response, and thrombosis (von Kugelgen, 2021; Zhang et al., 2024). The identified human P2Y receptor family consists of eight subtypes (P2Y_1_–P2Y_14_) (Coleman et al., 2021), which are widely expressed in endothelial cells and vascular smooth muscle cells and are important molecular hubs for maintaining cardiovascular homeostasis (Strassheim et al., 2020). Among them, P2Y_12_ receptor antagonists (such as clopidogrel and ticagrelor) have successfully achieved clinical transformation (Nappi, 2024), but the mechanism and application potential of other subtypes (such as P2Y_4_ and P2Y_11_) still need to be further explored (von Kügelgen, 2024).

Although a substantial body of literature has accumulated on the role of P2Y receptor in CVD_S_, systematic quantitative assessments and trend analyses remain limited. With the explosive growth in research literature production, the need for new approaches to structure knowledge has emerged. As emphasized by Kokol et al. (2021), bibliometrics provides a distinct advantage over traditional manual reviews by enabling the objective mapping of vast knowledge landscapes, minimizing subjective bias, and revealing hidden research fronts that qualitative synthesis might overlook (Kokol et al., 2021; Ng et al., 2025). Bibliometrics can delineate the development of a discipline by quantitatively analyzing literature distribution, collaboration networks, and knowledge evolution. Complementary to this, altmetrics provides a unique perspective for assessing societal attention, clinical translation potential, and public influence by tracking the dissemination and discussion of research across news, policy, and social media platforms (Garcia-Villar, 2021; Erdt et al., 2016).

However, relying on a single metric creates a “blind spot” in understanding the full trajectory of medical research. To date, no systematic study has integrated bibliometric and altmetric analyses for the specific topic of P2Y receptors in CVDs. This study is driven by the hypothesis that academic impact, measured by citations, diverges from social visibility, captured by Altmetrics, within P2Y research—a gap reflecting differing priorities between mechanistic exploration and public health. We therefore introduce a novel dual-metric framework integrating bibliometric and altmetric analyses. Moving beyond simple hotspot visualization with tools like CiteSpace and VOSviewer, we aim to validate this divergence, identify core translational barriers, and forecast emerging trends. This framework offers a new conceptual method for evaluating the translation of P2Y research from the laboratory to the societal domain.

## 2 Materials and methods

### 2.1 Data collection

To ensure comprehensive coverage of relevant literature, a systematic search was conducted across three academic databases and one altmetrics platform. The selected academic databases—Web of Science Core Collection (WoSCC), Scopus, and PubMed Central (PMC)—were chosen for their academic rigor and extensive coverage of the biomedical literature. Altmetric data, collected *via* Altmetric Explorer (www.altmetric.com), include the Altmetric Attention Score (AAS) and mentions across news outlets, social media platforms, and policy documents.

**FIGURE 1:**
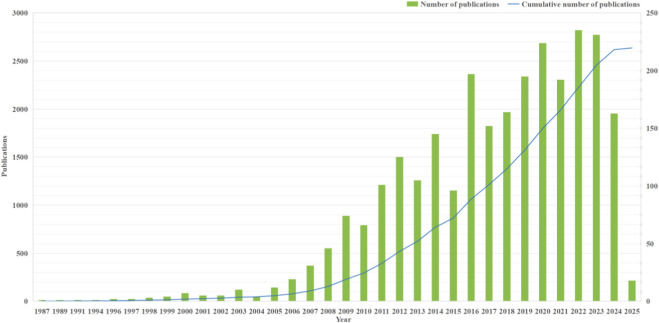
Annual and cumulative publications from 2005 to 2025.

The search strategy combined keywords related to “P2Y receptor” and “cardiovascular disease” and used wildcards and truncators to cover all variants, with P2Y-related terms: “P2Y,” “Purinergic P2Y Receptors,” “P2Y Purinoceptor” and cardiovascular disease related terms: “cardi” (covering “cardiology,” “cardiovascular,” “cardiac,” etc.) as topics. The search was limited to English articles and reviews published between 2005 and 2025 (up to 7 July 2025), and a total of 10,483 articles were retrieved. Two independent investigators (Mengyu Yang and Xuanrui Chen) screened titles and abstracts to exclude irrelevant studies (e.g., non-cardiovascular, non-human/non-clinical studies) and duplicate articles, and differences were resolved by consensus or consultation with third-party investigators (Jingchun Zeng), resulting in 2,591 articles included. This data is used to analyze and visualize authors, institutions, countries, journals, co-cited references, and keywords. Figure 1 illustrates the search and screening process.

### 2.2 Bibliometric analysis

Three primary tools were employed for bibliometric analysis. CiteSpace (version 6.4.1) (Chen, 2020) was used to construct author-institution collaboration networks and keyword co-occurrence maps, and to detect citation bursts for identifying emerging research frontiers. VOSviewer (version 1.6.20) (van Eck and Waltman, 2010) was utilized to visualize co-citation networks, national collaboration networks, and journal relationships. In these visualizations, node size corresponds to the number of publications, and connection thickness indicates the strength of collaboration. The Bibliometrix R package (Aria and Cuccurullo, 2017) was applied to quantify annual publication trends, journal distributions, and citation metrics, including total citations and the h-index.

### 2.3 Altmetric analysis

Data from Altmetric Explorer (www.altmetric.com) were used to obtain the Altmetric Attention Score (AAS) for each publication and to identify highly mentioned articles. We extracted AAS data and mention counts from various sources, including news outlets (e.g., The New York Times), social media platforms (e.g., Twitter/X), and policy documents to assess the broader societal impact of the research. The Spearman correlation coefficient was calculated to analyze relationships, and a *p*-value of <0.05 was considered statistically significant for assessing the validity of the observed societal impact.

## 3 Results

### 3.1 Annual and cumulative publications

**FIGURE 2:**
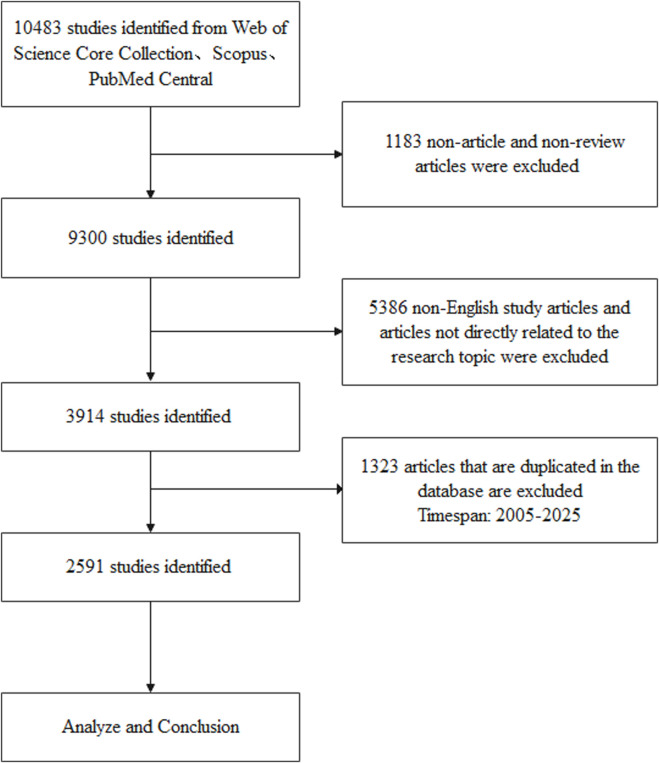
Flow chart of thesis collection and screening process.

The trend of annual and cumulative publications provides a key empirical basis for evaluating the current development of the field and predicting its future trend. As shown in Figure 2, the study of P2Y receptors in the CVDs has experienced three distinct growth phases between 2005 and 2025. The initial period, from 2005 to 2010, was the embryonic stage, marked by an explosive growth in annual publications, which surged from 12 to 248. This represented an impressive average annual growth rate of 60% and a 20-fold increase in cumulative publications, signifying a critical point of expansion that laid the groundwork for future advancements. The second phase, from 2011 to 2019, was one of steady expansion. During this time, the number of annual papers fluctuated but generally stabilized between 100 and 200, with the cumulative total surpassing 1,500. The research focus shifted from basic mechanisms to clinical translation. While the total volume expanded by 4.2 times, the average annual growth rate moderated to 22%, and the ratio of new papers to the existing stock decreased from 90% to 13%, indicating a shift from a rapid growth phase to stable development. The third and current phase, from 2020 to 2025, represents the mature period. The annual publication volume has been maintained at around 200 articles, with a slower annual growth rate of 11.6%. New research priorities have emerged, focusing on precision medicine and drug resistance mechanisms. Over this decade, the cumulative number of published papers increased by 148%, with a compound annual growth rate of approximately 10.5%. Although the figures for 2025 are not yet complete, strong growth is expected to continue. The continuous increase in both cumulative and annual publications powerfully demonstrates the growing academic vitality of P2Y receptor research in the CVDs field, attracting increasing attention from the scientific community.

### 3.2 Author and institutions analysis

VOSviewer 1.6.20 and CiteSpace 6.4.1 software were used to analyze the author collaboration network and institutional research pattern of P2Y receptors in the CVDs research field, including data from 12,292 authors and 4,653 institutions, revealing the core academic strength and global collaboration characteristics of this field.

#### 3.2.1 Author analysis: Core scholars and collaborative networks

The co-occurrence network of 174 authors with at least ten publications exhibits a “hub-and-spoke” topology centered on high-yield scholars (Supplementary Figure 1). Dominick J. Angiolillo, with 132 articles, is the leading contributor, representing 21.46% of the total output and forming the network’s core. Robert F. Storey and Deepak L. Bhatt are identified through centrality analysis as pivotal intellectual bridges, sustaining a transatlantic collaborative axis between institutions in the United States and Europe. Cluster analysis delineates three primary research domains: Angiolillo’s group concentrates on antithrombotic optimization after PCI, Storey’s team explores inflammatory mechanisms, and Bhatt’s cohort focuses on individualized treatment strategies (Supplementary Figure 2). This configuration demonstrates that the field is propelled by highly specialized, inter-institutional consortia rather than isolated investigators (Galli and Angiolillo, 2024; Angiolillo et al., 2009; Galli et al., 2022).

The international cooperation network in this field is characterized by a “hub-and-spoke” pattern, with the United States at the center and Europe and Asia as key branches. A prime example of this transatlantic collaboration is seen in the work of Robert F. Storey (University of Sheffield, UK), who co-authored 37 papers with Angiolillo’s team, advancing research on antiplatelet therapy (Bhatt et al., 2016; Capodanno et al., 2023). Cluster analysis showed Cluster #1 (red): Angiolillo as the core, focusing on clinical efficacy evaluation (such as optimization of antithrombotic regimen after PCI (Galli and Angiolillo, 2023; Lee et al., 2023)); Cluster #2 (blue): Storey as the core, focusing on inflammatory mechanism research (Thomas and Storey, 2015a; Parker and Storey, 2024) and Cluster #3 (green): Bhatt (Harvard Medical School, USA) as the core, focusing on individualized treatment (Bhatt et al., 2012).

#### 3.2.2 Institutional analysis: Global research patterns and interdisciplinary

The institutional cooperation network presents the characteristics of “leading by head institution and distinct regional characteristics.” The top ten institutions in terms of document volume are mainly distributed in the United States, Britain, China and Austria (Supplementary Table 1; Supplementary Figure 3).

Within the Asian network, Huazhong University of Science and Technology holds considerable regional influence. This institution guides the formulation of antithrombotic consensus for East Asian populations, highlighting distinctions in clinical outcomes by race and genotype (Li et al., 2017)). Consequently, the institutional landscape demonstrates clear geographic specialization: North American organizations lead guideline updates (Rao et al., 2025).

### 3.3 National distribution and cooperation analysis

The global research landscape comprises 68 countries and regions, organized within a highly centralized collaboration network (Supplementary Figure 4). The United States, with 941 articles, acts as the central hub for international cooperation. China (367 articles) and Italy (333 articles) serve as critical regional sub-centers within the Asian and European networks, respectively (Supplementary Table 2). The strategic partnership between China and the United States exemplifies the efficacy of such transnational collaboration. By integrating large-scale clinical cohorts from China with experimental design protocols from the United States, a joint study elucidated the molecular mechanisms of platelet activation in diabetic patients (Hu et al., 2017), Published in Circulation, this work demonstrates how cross-border synergy can accelerate the translation from basic target discovery to therapeutic optimization.

#### 3.3.1 Dynamic evolution of annual publication volume

The column chart generated with R’s ggplot2 package (Supplementary Figure 5) illustrates the annual publication output of the top ten countries, revealing several distinct trends: the United States consistently maintained a leading position, with annual publications stable between 150 and 200; China exhibited rapid growth, rising from fewer than 10 articles in 2005 to over 150 by 2020, surpassing several other nations, particularly after 2015; European countries such as Germany and the United Kingdom sustained steady outputs of 30–80 articles per year, whereas South Korea and the Netherlands demonstrated substantial growth in later years.

Overall, research on P2Y receptors in the field of cardiovascular diseases exhibits a global pattern characterized by “leadership from the United States, foundational contributions from Europe, and growing engagement from China, Japan, and South Korea.” Cross-regional collaboration is dominated by technology transfer from North America to Asia and by mechanistic research synergies within Europe, reflecting a close integration of basic and clinical investigation. Future efforts should encourage participation from emerging research regions such as Africa, including, for example, investigations into ethnic variations in P2Y receptor function initiated by Nigerian researchers. This differentiated developmental trajectory not only highlights the openness and vitality of the field, but also offers diverse perspectives and collaborative opportunities for global cardiovascular disease prevention and control.

### 3.4 Analysis of journals

This study employed VOSviewer to analyze the journal sources of published literature and co-cited references, thereby identifying the core academic platforms and influence of P2Y research within the field of CVD_S_.

#### 3.4.1 Distribution of journal publications

The analysis identified 2,591 articles published across 573 journals; the top 10 most productive journals are listed in Supplementary Table 3. Thrombosis and Haemostasis ranked first with 72 publications, accounting for 2.78% of all articles in this domain. Together, the top ten journals published 482 articles. Eight of these journals are classified as JCR Q1, and seven possess an impact factor greater than 5. The Journal of the American Heart Association, which has the highest impact factor (63.5, Q1), illustrates that research in this field is predominantly disseminated in high-impact cardiovascular disease journals.

#### 3.4.2 Analysis of co-cited journals

Co-citation analysis revealed that the top five journals each accumulated over 3,000 citations (Supplementary Table 4), with the Journal of the American College of Cardiology ranking first at 8,352 citations, followed by Circulation (8,202 citations) and The New England Journal of Medicine (7,381 citations). Nine of the top ten co-cited journals belong to the JCR Q1 quartile, eight possess an impact factor exceeding 5, and The Lancet has the highest impact factor at 98.4. These findings underscore the central role of high-impact journals in both foundational research and the formulation of clinical guidelines.

#### 3.4.3 Characteristics of interdisciplinary research

The double-map overlay of journals generated by CiteSpace (Supplementary Figure 6) illustrates a multidisciplinary citation pathway linking the journal clusters on the left with those on the right:

The “MOLECULAR, BIOLOGY, IMMUNOLOGY-MOLECULAR, BIOLOGY, GENETICS” pathway captures a pivotal transition in P2Y receptor research, shifting focus from classical immunological processes—such as inflammation mediated by P2Y receptors (Zerr et al., 2011; Degagné et al., 2012; Klaver and Thurnher, 2021)—toward genetic regulation, exemplified by the use of knockout techniques to resolve gene expression networks (Dales et al., 2025).This progression has led to a “signal-gene” network model, which originated from the growing demand for receptor subtype specificity.

The “MEDICINE, MEDICAL, CLINICAL → HEALTH, NURSING → MEDICINE” citation pathway illustrates how clinical medicine and nursing practice refine antithrombotic strategies and precision nursing in P2Y receptor-targeted therapy for CVDs by focusing on the efficacy, safety, and individualized use of P2Y receptor-related medications (Lopes et al., 2019; Kirchhof et al., 2014; Gopinath Subramaniya, 2021). This approach lowers the risk of cardiovascular adverse events while fostering integration across clinical diagnosis, nursing, health management, and personalized medicine.

Research in this area appears primarily in high-impact journals dedicated to CVDs, with highly cited publications concentrating on clinical guidelines and foundational studies, reflecting a distinctly interdisciplinary orientation. Journal analysis offers valuable guidance for researchers choosing suitable publication venues, tracking emerging advances, and pursuing cross-disciplinary collaboration.

### 3.5 Keyword co-occurrence and burst analysis

Keyword co-occurrence and burst detection analyses delineated the core themes, evolving hotspots, and emerging trends in P2Y receptor research related to CVDs. The keyword co-occurrence network generated by CiteSpace comprises 395 nodes, with node size proportional to frequency of occurrence. Nodes with a purple outer ring denote high betweenness centrality, while those highlighted in red represent keywords with citation bursts (Supplementary Figure 7).

#### 3.5.1 High-frequency keywords and research hotspots

Clopidogrel (1,168) was the most frequently occurring keyword, followed by other high-frequency terms such as percutaneous coronary intervention (1,046) and myocardial infarction (604), underscoring the continued importance of conventional antiplatelet therapy in acute coronary syndrome. Within the keyword co-occurrence network, high-degree terms including “bleeding,” “antiplatelet drug,” and “drug dose” reveal the evolving clinical focus of P2Y receptor research: from establishing the efficacy of targeted therapy in myocardial infarction, to addressing interindividual variability in receptor inhibition through dose adjustment, to managing side effects such as bleeding, and ultimately toward balancing individual patient outcomes.

#### 3.5.2 Keyword clustering and topic evolution

The keyword co-occurrence network is partitioned into eight clusters by the LSI algorithm (Supplementary Figure 8), with a modularity Q value of 0.48 (>0.3), indicating a well-defined thematic structure. Cluster #1 (Purinergic P2Y_12_): The core study (Hochholzer et al., 2017) focused on optimizing the timing of antiplatelet therapy and demonstrated that administering prasugrel during cangrelor infusion avoids the transient platelet suppression associated with conventional methods—loading P2Y_12_ inhibitors after cangrelor discontinuation—providing a safe and effective alternative. Cluster #4 (CYP2C19 polymorphism): The core study (Xi et al., 2020) used real-world large-sample data to show, for the first time, that ticagrelor reduces ischemic events by 51% compared to clopidogrel in patients at high risk for CYP2C19 loss-of-function, offering critical evidence for genotype-guided antiplatelet therapy. Cluster #6 (Dual antiplatelet therapy, DAPT): The core study (Badri et al., 2017) found that the P2Y_12_ inhibitor component of preoperative DAPT significantly increases surgical delay and perioperative bleeding risk in NSTEMI patients requiring early CABG, thereby challenging current guideline recommendations for early intensive antiplatelet therapy. The high Modularity Q (0.48) statistically confirms that these clusters are not random aggregations but distinct, well-structured thematic sub-domains within the P2Y research landscape.

#### 3.5.3 Emergent keywords and the evolution path of research topics

The chronological shift and thematic evolution of research hotspots were visualized with CiteSpace (Supplementary Figure 9) and a Sankey diagram (Supplementary Figure 10). Over the past two decades (2005–2025), research on P2Y receptors in cardiovascular diseases has progressed through three distinct stages. The initial stage (2005–2012) involved exploring pharmacological mechanisms, as defined by early high-frequency keywords such as “clopidogrel resistance” and “adenosine diphosphate.” The prominence of these terms reflects a foundational phase focused on elucidating the metabolic pathways of early P2Y12 inhibitors. A second stage (2013–2018) emphasized structural optimization and precision intervention, marked by the emergence of thiophenes and their analogues, which shifted the focus from mechanism identification to compound refinement. The third stage (2019–2025) is characterized by individualization and clinical strategy integration, exemplified by “dual antiplatelet therapy,” which achieved the highest emergent strength (46.85). This stage confirms a paradigm shift toward regimen optimization, a surge that correlates with recent guideline updates and suggests evolving clinical consensus drives bibliographic output (Cuisset et al., 2017; Lone and Khan, 2020; Vrints et al., 2024; Rao et al., 2025).

This evolutionary trajectory is statistically corroborated by keyword clustering results (modularity Q = 0.48), which identified three major thematic clusters: “P2Y_12_ inhibitor therapy optimization,” “gene polymorphism and individualized medication,” and “dual antiplatelet therapy (DAPT) risk control.” Collectively, these findings highlight the transition of P2Y receptors from theoretical targets to strategic pillars in precision medicine.

### 3.6 Analysis of highly co-cited literature

In this study, we employed CiteSpace to perform a co-citation analysis of 2,637 included references, tracing the evolution of landmark research and knowledge pathways related to P2Y receptors within the CVD_S_ research field. The resulting co-citation network comprises 690 nodes, 1,278 links, and five clusters (Supplementary Figure 11; modularity Q = 0.7476, mean silhouette S = 0.8915), reflecting a well-defined clustering structure and high thematic coherence.

#### 3.6.1 Core co-cited literature and academic contributions

Ten key articles were identified through emergent analysis (Supplementary Figure 12). The study with the highest emergent intensity (62.64) (Wallentin et al., 2009), serves as a critical structural turning point. This exceptional burst strength quantitatively demonstrates how the PLATO trial fundamentally restructured the citation network, shifting the academic consensus from clopidogrel to ticagrelor. Similarly, subsequent high-intensity research (Mehran et al., 2019) marks the onset of a “de-escalation” paradigm. These high-burst nodes act as intellectual bridges, guiding the field’s evolution from aggressive antithrombotic regimens toward safety-prioritized monotherapy.

#### 3.6.2 Topic clustering and evolution of co-introduction networks

The co-citation network divides into five clusters (Supplementary Figure 13), reflecting thematic research relevance: Cluster #0, novel platelet ADP receptor inhibition, focuses on advances in applying new P2Y_12_ receptor inhibitors for treating CVDs, tracing a research pathway from mechanistic exploration and drug development to clinical translation; Cluster #1, crushed ticagrelor, reflects scholarly attention to optimizing P2Y_12_ inhibitor administration in ACS patients, particularly emphasizing how alterations to ticagrelor’s formulation and onset speed advance individualized treatment, bridging fundamental research and clinical practice; Cluster #2, P2Y_12_ inhibitor therapy, centers on monotherapy strategies, illustrating the shift in antiplatelet treatment from intensive combination toward precision medicine, and highlighting its evolving role from adjunctive to central; Cluster #3, high on-treatment platelet reactivity (HTPR), addresses individual variability in platelet reactivity and P2Y_12_ inhibitor resistance, underscoring the importance of phenotypic and genotypic testing in risk stratification and therapy optimization, which underpin the development of tailored antithrombotic pathways; Cluster #4, anticoagulant therapy, focuses on individualized adjustment of antithrombotic strategies, especially “de-escalation therapy” options post-PCI, and emphasizes precision dosing guided by ethnic sensitivity and functional testing, facilitating a transition from uniform intensive treatment toward risk-balanced strategies.

### 3.7 Highly cited and high-AAS literature: Altmetric analysis and potential confounders

To comprehensively capture the diversity of knowledge on P2Y in cardiovascular disease (CVD) and to counterbalance the predominance of clinical studies among the most-cited works, we implemented a tiered screening strategy. This process selected the ten most representative, highly cited articles (each with ≥160 citations, Table 1) from distinct research categories, comprising four clinical studies, four basic or translational studies, one guideline, and one narrative review. This selection essentially spans the complete knowledge chain from receptor biology and pharmacogenomics to therapeutic decisions and normative documents. We also analyzed the top ten articles ranked by Altmetric Attention Score (AAS) Supplementary Table 5. The results show that over half of the high-AAS articles were published between 2019 and 2023, primarily addressing the optimization of P2Y_12_ receptor inhibitor use in acute coronary syndrome and other high-risk contexts. Notably, a study led by Lars Wallentin appeared on both the high-citation and high-AAS lists, achieving an AAS of 658 and thereby demonstrating a dual performance of high academic impact and broad societal attention.

**TABLE 1:** Information table of 10 highly cited literatures.

Type	Study	Journal	Citation	AAS	Highlights
Clinical Research	Wallentin et al. (2009)	New England Journal of Medicine	5,585	659	In a multicenter RCT of 18,624 patients with acute coronary syndrome (ACS), ticagrelor demonstrated a significant reduction in patients ‘risk of the primary composite endpoint (cardiovascular death, myocardial infarction, or stroke) at 12 months compared with clopidogrel and a simultaneous reduction in all-cause mortality. There was no difference in overall major bleeding rates between the two groups, but ticagrelor had a higher risk of non-coronary bypass related major bleeding.
	Antman et al. (2008)	Journal of the American College of Cardiology	738	27	A landmark analysis based on the TRITON-TIMI 38 trial showed that prasugrel loading and maintenance doses significantly reduced ischemic events (myocardial infarction, stent thrombosis, etc.) in patients with ACS undergoing percutaneous coronary intervention (PCI), with a net clinical benefit superior to clopidogrel in the early (0–3 days) and long-term (3 days to endpoint) periods. It should be noted that the risk of bleeding increases mainly in the maintenance dose phase, suggesting that maintenance dose strategies need to be optimized for high-risk populations such as the elderly and low-weight individuals.
	Mehran et al. (2019)	New England Journal of Medicine	737	380	The TWILIGHT trial, which included 7119 PCI patients at high bleeding/ischemia risk, showed that switching to ticagrelor monotherapy after 3 months of dual antithrombotic therapy (DAPT) significantly reduced the risk of clinically relevant bleeding within 12 months without increasing the composite endpoint of death, myocardial infarction, or stroke, providing key evidence for a step-down strategy for antithrombotic therapy.
	Price et al. (2008)	European Heart Journal	557	10	A total of 380 patients with DES were studied. VerifyNow P2Y_12_ test showed that patients with platelet hyperreactivity after clopidogrel treatment had a significantly increased risk of cardiovascular death and stent thrombosis within 6 months. It was confirmed that platelet hyperreactivity was an independent predictor of thrombotic events in this population, providing a basis for individualized antithrombotic strategy adjustment in high-risk patients.
Basic Research	Jennings (2009)	Thrombosis and Haemostasis	357	1	Studies have pointed out that platelets mediate hemostasis and pathological thrombosis in vascular injury. Although existing antiplatelet drugs (aspirin +P2Y_12_ antagonist) can target thromboxane A_2_/ADP pathway, they cannot inhibit thrombin activation, resulting in high risk of residual thrombosis and associated bleeding risk. Novel protease-activated receptor-1 (PAR-1) inhibitors synergistically inhibit the thrombin pathway without interfering with physiological hemostasis, opening new avenues for optimizing antithrombotic strategies.
	( Zhang et al., 2014 )	Nature	326	67	This study was the first to resolve the high-resolution crystal structure (2.6 Å) of the human-derived P2Y_12_ receptor-antagonist AZD1283 complex, revealing its unique vertical helical V conformation and dynamic disulfide bond characteristics, defining the binding site of non-nucleoside ligands and proposing a double-pocket dinucleotide binding model. This structure provides a key structural basis for the development of a new generation of P2Y_12_ receptor-targeted drugs, which is expected to overcome the clinical limitations of existing antithrombotic drugs (such as clopidogrel with a long half-life and ticagrelor side effects).
	Chen et al. (2010)	Science Signaling	181	1	It was found that neutrophils stimulated by bacterial peptides release ATP through panx1 half-channel and co-locate with P2Y_2_ receptors on cell surface to form purinergic signaling system. This system significantly enhances neutrophil activation *via* FcγR/IL-8R/C5aR/LTB4R mediated ATP-P2Y_2_ autocrine loop. Inhibition of panx1 or silencing of P2Y_2_ receptors blocks this signaling pathway and impairs the host innate immune response, confirming that purinergic signaling is the fundamental mechanism of neutrophil activation and antimicrobial defense.
	Wang et al. (2015)	Journal of Clinical Investigation	160	64	The P2Y_2_ receptor-Gq/G11 protein axis in endothelial cells has been confirmed to be the central mechanism for shear force sensing: shear force triggers ATP release → activation of P2Y_2_ receptors, mediating calcium transient/eNOS activation/vasodilation. In experiments, mice with endothelium-specific knockouts of P2Y_2_ or Gq/G11 lost flow-mediated vasodilation, reduced eNOS activity and developed hypertension, suggesting that this pathway maintains blood pressure homeostasis by regulating basal NO production.
Guidelines	Levine et al. (2016)	Journal of the American College of Cardiology	1,497	581	The guidelines emphasize that clinical decision-making for dual antiplatelet therapy (DAPT) needs to be individualized: 6–12 months of basic treatment is recommended, patients with DAPT score ≥2 may benefit from extended treatment; ticagrelor is preferred for patients with acute coronary syndrome. Aspirin should be administered at low doses (75–100 mg/d, preferably 81 mg/d) to optimize the antithrombotic strategy. This framework significantly improves the balance of ischemia and bleeding risk
Review	Abbracchio et al. (2006)	Pharmacological Reviews	1,198	12	This review systematically summarizes the progress of P2Y receptor, including: more subtypes have been cloned, characterized and de-orphaned, laying a foundation for subtype classification; in-depth exploration of signaling mechanisms (including ion channel interaction), selective agonist/antagonist design, nucleotide release and extracellular enzymatic hydrolysis mechanisms; new discoveries involving receptor interaction, transcriptional regulation, tissue distribution and function, and continuous expansion of therapeutic potential, providing a comprehensive reference for research in this field.

Spearman correlation analysis elucidated the relationships between the AAS and its constituent attention dimensions. The strongest correlations were between AAS and mentions on platform X (*r* = 0.5957, *p* < 0.001) and news mentions (*r* = 0.5441, *p* < 0.001), indicating that social media and news coverage were the primary drivers of the Altmetric score in this sample. Moderate correlations existed with clinical guideline citations (*r* = 0.3984, *p* < 0.001), Dimensions citations (*r* = 0.3743, *p* < 0.001), and Mendeley readership (*r* = 0.3705, *p* < 0.001). Positive correlations of moderate strength were also observed for Facebook and blog mentions (*r* ≈ 0.35, all *p* < 0.001). In contrast, policy document mentions, patent citations, Wikipedia mentions, and F1000 mentions showed weaker correlations with AAS. Correlations between AAS and Weibo mentions or peer-review mentions were negligible, while Reddit mentions showed no significant correlation. This pattern indicates that for P2Y-related cardiovascular research, the Altmetric signal is driven primarily by English-language social and news media, which subsequently influences more traditional evaluation systems through guideline and academic citations.

Several potential confounding factors must be considered when interpreting the only moderate correlation between AAS and citation-based metrics, though they are only briefly highlighted here. First, publication eras differ; many highly cited basic science and pharmacogenomic studies were published before social media was prevalent or Altmetric coverage was comprehensive, making their online attention unlikely to match their long-term citation accumulation. Second, journal influence and its dissemination resources can affect AAS, as high-impact journals often amplify a study’s online visibility through press releases. Third, Altmetric primarily captures the English-language digital ecosystem, with limited coverage of non-English regions. These factors collectively complicate the establishment of a simple linear relationship between AAS and traditional citations.

In summary, thematic differences between highly cited and high-AAS literature remain evident. High-AAS literature concentrates more on issues with direct clinical or public health implications, such as comparisons between P2Y_12_ receptor inhibitors, antiplatelet regimen optimization in acute coronary syndrome, and safety signals regarding antithrombotic drug interactions during the COVID-19 pandemic. These topics are more prone to trigger media coverage and social media discussion. Highly cited literature, conversely, focuses more on P2Y receptor biology and signal transduction, receptor structure elucidation, pharmacogenomic evidence for drug response, and evaluations of long-term antithrombotic strategies. The former reflects translatable topics with high dissemination potential, while the latter constitutes the structural foundation of domain knowledge. Together, they delineate the distinct evolutionary trajectories of P2Y receptor-related research along the dual pathways of academic citation and societal discussion.

## 4 Discussion

This study employs bibliometric and altmetric analyses to chart the evolution of P2Y receptor research in cardiovascular disease. Our dual-metric approach uncovers a structural transition from a narrow focus on P2Y_12_-mediated antithrombosis toward a complex network encompassing immune inflammation and tissue regeneration. The keyword “burst” for non-P2Y_12_ subtypes in Phase II of our trend analysis underscores this growing recognition of functional heterogeneity. This diversification is driven by the distinct roles of individual subtypes in cellular specificity and signaling crosstalk. For example, the P2Y_12_ receptor promotes thrombosis through ADP-mediated platelet activation, and pharmacological inhibition of this pathway, including agents such as ticagrelor, has been extensively applied in the post-ACS and post-PCI setting (Angiolillo et al., 2017); Emerging research, however, highlights divergent pathways for other subtypes. The P2Y_6_ receptor exacerbates atherosclerosis by activating the NLRP3 inflammasome in macrophages (Collado and Zhou, 2025), while the P2Y_2_ receptor fosters myocardial regeneration by modulating cardiac progenitor cell proliferation through the Hippo pathway (Khalafalla et al., 2017).

### 4.1 From “P2Y_12_ inhibition” to precision antithrombotic strategies: Keywords, bursts, and co-citation evidence

Cross-interpretation of keyword co-occurrence clusters, burst dynamics, and co-citation clusters indicates that thematic differentiation in P2Y-related cardiovascular research is driven primarily by clinical decision-making challenges. In the co-occurrence network, high-frequency terms such as clopidogrel, PCI, and myocardial infarction have long occupied a central position. Concurrently, terms with high-degree connectivity, including bleeding, drug dose, and DAPT, have risen in prominence. This pattern suggests a shift in research focus from verifying efficacy and feasibility toward the refined optimization of administration timing, dosage selection, and population stratification.

LSI clustering and timeline analyses further delineate this transition through three progressive themes: (1) procedural optimization of P2Y_12_ inhibitor timing and switching, which addresses practical challenges like oral loading during cangrelor bridging and avoids “efficacy gaps” in urgent or perioperative scenarios; (2) response variability and stratified therapy, exemplified by CYP2C19 testing and high on-treatment platelet reactivity (HTPR); and (3) strategic rebalancing focused on bleeding risk control and duration optimization within dual antiplatelet therapy (DAPT). Temporally, DAPT demonstrated the strongest citation burst (strength: 46.85) from 2019 to 2025, marking it as a primary recent growth area. The co-citation network corroborates this by revealing a structural shift in the field’s knowledge base from “establishing consensus through landmark trials” toward “regimen adjustment under safety constraints.” Specifically, the PLATO trial, the node with the highest burst strength (62.64), corresponds to the transition from clopidogrel-based regimens to more potent P2Y_12_ inhibition with ticagrelor. Subsequently, high-burst studies focusing on “de-escalation” and “monotherapy” have formed a bridging theme that links the evidence chains for “efficacy enhancement” and “bleeding control,” thereby reflecting ongoing debate on risk-stratification-guided antithrombotic strategies.

Furthermore, the “short-term DAPT—de-escalation/monotherapy—stratified decision-making” axis identified through this cross-interpretation is not an isolated network phenomenon but corresponds directly to recent clinical research and evidence updates. The SHARE randomized trial showed that switching from DAPT to P2Y_12_ inhibitor monotherapy 3 months after PCI was non-inferior to 12-month DAPT with respect to NACE, supporting the viability of a strategy involving shortened combination therapy followed by P2Y_12_ inhibitor monotherapy (Min et al., 2024). Systematic reviews and meta-analyses in ACS populations further demonstrate that P2Y_12_ inhibitor monotherapy after short-term DAPT significantly lowers bleeding risk without increasing ischemic events, while also hinting at potential stratified benefits between different agents, such as ticagrelor *versus* clopidogrel (Galli et al., 2024).

In individualized medication, CYP2C19 genotyping—especially for loss-of-function [LOF] carriers—and high on-treatment platelet reactivity (HTPR) assessments guide the escalation or de-escalation of P2Y_12_ inhibitor therapy. For patients carrying LOF alleles or exhibiting HTPR alongside high ischemic risk, switching from clopidogrel to ticagrelor or prasugrel is preferred to mitigate ischemic events. Conversely, for patients with lower ischemic but higher bleeding risk, de-escalation from potent P2Y_12_ inhibitors to clopidogrel or shortening dual antiplatelet therapy (DAPT) duration after the acute phase is considered, thereby operationalizing the “CYP2C19–HTPR” framework into stratified treatment pathways (van den Broek et al., 2024). A 2025 individual participant data meta-analysis in the BMJ further compared P2Y_12_ inhibitor monotherapy with aspirin monotherapy as maintenance therapy after DAPT discontinuation, reinforcing the re-evaluation of long-term strategies as a current focus (Giacoppo et al., 2025).

The convergent trajectory from co-occurrence, burst detection, and co-citation analyses indicates that research hotspots are increasingly centered on actionable clinical decision points, such as duration selection, medication bridging, and stratified decision-making. These themes appear in bibliometric maps as highly connected terms and high-burst keywords, corresponding to points of divergence frequently debated in recent trials and reviews. This alignment offers a reference for more refined subsequent evidence synthesis and clinical study design.

### 4.2 The dual role and subtype specificity of P2Y receptors in the regulation of immune inflammation in CVD_S_

The imbalance between inflammation and immune response constitutes a central mechanism in the development of CVDs (Wang Z. et al., 2025; Gupta et al., 2023). P2Y receptors detect extracellular nucleotides such as ATP and ADP, thereby contributing to immune cell activation and inflammatory cascades (Le Duc D et al., 2017; Thomas and Storey, 2015b),although their functions are highly subtype-specific and context-dependent (Klaver and Thurnher, 2021).

The mechanisms of pro-inflammatory subtypes are well-established: P2Y_1_, P2Y_2_, P2Y_6_, and P2Y_14_ primarily promote inflammation. The P2Y_1_ receptor enhances TNFα-induced vascular inflammation by upregulating leukocyte adhesion molecule expression in endothelial cells (Zerr et al., 2011). Activation of the P2Y_6_ receptor stimulates the release of pro-inflammatory factors such as IL-6 and TNF-α *via* the macrophage NLRP3 inflammasome and accelerates atherosclerosis (Collado and Zhou, 2025); The P2Y_14_ receptor is activated by UDP-G and amplifies neutrophil infiltration through chemokine-mediated recruitment (Zhang et al., 2024). These subtypes collectively drive disease progression through a cascading process involving “injury signal detection–inflammatory mediator release–pathological amplification”.

The function of anti-inflammatory and regulatory subtypes is reflected in their maintenance of immune homeostasis; P2Y_11_ receptors interact with the IL-1R pathway to promote macrophage M2 polarization and participate in tissue repair (Klaver and Thurnher, 2023). P2Y_12_ exhibits a particularly typical dual role, as its activation induces platelets to release P-selectin, thereby exacerbating inflammation (Lee et al., 2023).Antagonists such as ticagrelor reduce inflammatory responses by inhibiting platelet–leukocyte interactions (Mansour et al., 2020), which depend bidirectionally on the pathological stage and cellular microenvironment (Xiao et al., 2024).

A key bottleneck in current research is the lack of clinically available, highly selective modulators for most receptor subtypes, and differences in expression between animal models and humans limit the extrapolation of results. Future studies should integrate single-cell sequencing and spatial transcriptomic technologies to analyze the spatiotemporal expression patterns of P2Y receptors within specific cellular subsets, thereby providing a foundation for precise anti-inflammatory interventions.

### 4.3 P2Y receptor-mediated transcellular signaling network for myocardial protection and regeneration

Throughout myocardial ischemia/reperfusion injury and post-infarction repair, P2Y receptors function as a signaling hub coordinating the entire “injury–repair–regeneration” continuum by mediating cross-talk among cardiomyocytes, immune cells, and endothelial cells (Tobin et al., 2020; Djerada et al., 2017). In myocardial protection, receptor subtypes including P2Y_12_, P2Y_2_, P2Y_4_, and P2Y_14_ play pivotal roles. The P2Y_12_ receptor has attracted particular research interest due to its central position within platelet–immune–vascular networks (Puthanveedu et al., 2025). As a key mediator of ADP-induced platelet aggregation, P2Y_12_ antagonists such as clopidogrel reduce thrombosis and exert cardioprotective effects in myocardial infarction and ischemia/reperfusion injury (Zhang et al., 2017). P2Y_12_ expression in vascular smooth muscle and endothelial cells also modulates inflammatory mediators such as MCP-1, thereby contributing to vascular inflammation (Satonaka et al., 2015). Although direct evidence for a role in myocardial regeneration is lacking, the synergistic anti-inflammatory and antithrombotic effects of P2Y_12_ inhibition indirectly support cardiac repair, particularly given the influence of inflammation on regenerative capacity (Li et al., 2023).

The P2Y_14_ receptor recognizes UDP-glucose and induces neutrophil polarization toward a proinflammatory (N1) phenotype; its antagonist PPTN promotes a shift to the anti-inflammatory (N2) phenotype, thereby attenuating myocardial inflammation (Zhang et al., 2024). indicating its potential cardioprotective value. As a UTP-activated receptor subtype, P2Y_4_ demonstrates cardioprotective potential in myocardial ischemia/reperfusion injury (I/R): its activation is closely linked to intracellular Ca^2+^ dynamics, influences tissue repair *via* endothelial cell function and cardiac progenitor cell activity (Djerada et al., 2017), and may contribute to post-ischemic repair by regulating cell proliferation, migration, and anti-apoptotic mechanisms (Rafehi et al., 2017).

The role of P2Y_2_ receptors in myocardial protection and regeneration has been experimentally validated and holds direct therapeutic promise. Studies using *in vitro* models, mouse experiments, and isolated heart perfusion demonstrate that both UTP and the P2Y_2_R agonist MRS 2768 significantly reduce myocardial infarct size following I/R injury by activating P2Y_2_ receptors, improving cardiac function in a dose-dependent manner (Li et al., 2016; Cohen et al., 2011). These findings underscore its critical role in preserving cardiomyocyte integrity and function and countering ischemic injury (Khalafalla et al., 2017). Furthermore, P2Y_2_ receptors act as signaling nodes that support cardiomyocyte survival, endothelial cell function, and cardiac progenitor activation through regulation of intracellular Ca^2+^ concentrations and ERK1/2 pathways (Chang et al., 2008; Muscella et al., 2003; Weisman et al., 2001). However, P2Y_2_ also mediates fibrogenic responses in fibroblasts, suggesting a dual role in regenerative regulation (Braun et al., 2010), that warrants further refinement of targeting strategies.

In summary, P2Y receptors do not operate as isolated pathway components in myocardial protection and regeneration, but rather function as signaling hubs integrating multiple cell types. Their actions exhibit marked subtype specificity, cell-type dependency, and stage-dependent variation across pathology, forming a coordinated signaling network throughout the process of “damage–inflammation–repair–regeneration.” Future studies should prioritize delineating the spatiotemporal expression patterns of P2Y receptors across distinct cellular subsets to enable the development of precise interventional strategies.

### 4.4 Progress in clinical transformation and individualized treatment

Our co-citation analysis identified specific “structural turning points” that have reshaped clinical practice. The high burst strength of landmark studies, such as the PLATO trial, quantitatively marks the transition from clopidogrel to ticagrelor. This shift established P2Y12 antagonists as a key therapeutic strategy for the secondary prevention of atherosclerotic cardiovascular events (Angiolillo et al., 2017). The subsequent rise of “genotype-guided therapy” clusters (Cluster #4) reflects the integration of CYP2C19 testing into precision medicine (Al-Abcha et al., 2022). A current “de-escalation” shift is exemplified by the TROPICAL-ACS study, which demonstrated that a guided step-down strategy reduces bleeding events without elevating ischemic risk (Sibbing et al., 2017). A contemporary “de-escalation” shift is now underway, evidenced by the high centrality of studies focusing on bleeding risk reduction. Recent evidence from the TACSI study (Jeppsson et al., 2025). However, critically reminds us that intensified strategies are not universally applicable. This aligns with our bibliometric finding that “bleeding risk” and “individualization” have become dominant high-frequency keywords, driving the transition from “the more, the better” to “less is more” in antithrombotic strategy.

Significant progress has also been made in elucidating the X-ray crystal structures of small molecule ligands bound to both P2Y_1_ and P2Y_12_ receptors, while a cryo-electron microscopy structure of the P2Y_2_–Gq protein complex has further revealed the molecular basis of ligand recognition and signal transduction (Lan et al., 2025). Fluorescent imaging probes, high-affinity radioligands, and high-throughput screening methods have substantially enhanced the resolution of P2Y receptor activation and antagonist binding kinetics (Stéen et al., 2025). These structural and technological advances provide a foundation for the precise modulation and targeted drug design of P2Y_1_, P2Y_2_, P2Y_6_, and other subtypes, facilitating the development of more selective and safer P2Y receptor-based therapeutics with expanded potential in cardiovascular disease (Puthanveedu et al., 2025). As structural analysis and screening technologies continue to advance, novel P2Y receptor modulators may integrate genotyping, functional phenotyping, and disease stratification strategies, thereby shifting the paradigm from single-target inhibition toward precise targeting and individualized intervention, and offering more tailored treatment options for diverse cardiovascular conditions.

### 4.5 Interdisciplinary trends and technological integration

In recent years, research on P2Y receptors within the field of CVDs has exhibited a notable trend toward interdisciplinary integration, centered on incorporating nano-delivery systems, multi-omics analysis, artificial intelligence modeling, and real-time monitoring into drug development and functional regulation. Advances in materials and biotechnology have enabled Guo et al. (2024) to develop P2Y_12_-overexpressing cell membrane-coated nanoparticles that reverse the effects of antiplatelet drugs *in vivo*; Qin et al. (2025) outlined a strategy using micro-nano motors to assist targeted delivery of P2Y_6_ antagonists to atherosclerotic plaques, offering a novel approach for precise drug transport. In drug screening and mechanistic studies, Han et al. (2023) and Zhao et al. (2024) performed virtual screening based on domain rigidity and receptor scaffold design for P2Y_14_ and P2Y_6_, respectively, facilitating the identification of high-affinity compounds. Multi-omics and sensing technologies further support the elucidation of functional mechanisms and individualized monitoring: Collado and Zhou (2025) uncovered signaling pathways mediated by P2Y_6_ that promote foam cell formation, providing systematic validation for target identification. Dambruoso et al. (2024) employed thromboelastography (TEG) to monitor intraoperative bleeding risk associated with P2Y_12_ inhibitors, while Ahmad et al. (2025) developed a wearable sensor for real-time assessment of platelet activation. Artificial intelligence models also enhance personalized treatment approaches: Ortega-Paz et al. (2024) predict efficacy based on eGFR, Lin et al. (2025) integrated genetic data and coagulation parameters to construct a dose optimization system. Moreover, multi-scale modeling has revealed synergistic effects of P2Y_1_/P2Y_12_ signaling in thrombus stability (Bouchnita and Volpert, 2024; Patel et al., 2025), and implicated P2Y receptors in inflammation-thrombosis crosstalk (Seung et al., 2022) and vascular homeostasis regulation (Chen et al., 2024; Daghbouche-Rubio et al., 2024), opening new avenues for therapeutic expansion. Current challenges include limited subtype selectivity (Han et al., 2023) and interference from receptor heterodimerization (Puthanveedu et al., 2025). Future breakthroughs will require deeper integration of emerging technologies with P2Y receptor-specific regulatory strategies to achieve dynamic, stratified, and controllable intervention of P2Y signaling across disease states.

### 4.6 Discussion on the differences in focus between the academic community and society

Based on the joint analysis of Altmetric and citation metrics, this study reveals a significant disparity between the academic community and the general public in their attention to the same body of knowledge within P2Y receptor-related cardiovascular research. As shown in Supplementary Figure 14, the Altmetric Attention Score (AAS) correlates more strongly with activity on platform X and news coverage than with traditional citation metrics, demonstrating only a moderate positive correlation with citation counts and Mendeley readership. This pattern indicates that Altmetric primarily captures online attention centered on English-language social media and news outlets, rather than the academic impact traditionally reflected by cumulative citations. The observed “correlated but non-overlapping” relationship does not signify the superiority of one evaluation system over another but results from several intertwined structural factors. First, publication date directly influences the misalignment between academic and societal attention. Many highly cited foundational and translational studies were published before the rise of social media and systematic Altmetric data collection. While these works hold significant positions in traditional citation databases, they correspondingly lack societal resonance in terms of AAS. Conversely, more recent clinical and translational studies often benefit from multi-channel dissemination, such as journal press releases and official social media promotions, allowing them to achieve high AAS rapidly despite being in early stages of citation accumulation. Second, the dissemination capacity of the publishing journal itself is a critical variable. High-impact cardiovascular journals typically employ professional media teams and maintain active social media channels to proactively promote papers with clinical or public health significance. Consequently, studies of comparable academic merit tend to achieve greater Altmetric prominence when published in such venues. In contrast, studies published in highly specialized or lower-impact journals may be undervalued in Altmetric assessments despite reporting groundbreaking findings in areas like receptor mechanisms or pharmacogenomics, due to their limited dissemination resources. Furthermore, an inherent dissemination bias within the Altmetric system itself cannot be overlooked. Its scoring relies heavily on activity from English-language social platforms and news outlets, while discussions from non-English regions or specific online communities are less represented, thereby concentrating “global attention” along linguistic and geographical lines and amplifying evaluation imbalances.

After identifying these confounding factors, comparing high-AAS literature with highly cited literature provides a more nuanced understanding of the differing focus between academic and societal attention. High-AAS literature predominantly addresses topics with direct implications for clinical decision-making or public health, such as studies on different P2Y_12_ receptor inhibitors, antiplatelet strategies in acute coronary syndromes, and drug interactions in the context of emerging infectious diseases. This type of research is more likely to be covered by news media and reinterpreted by clinicians, patients, and policy groups on social media, thus holding an inherent advantage within the dimensions constituting the AAS. In contrast, highly cited literature more frequently addresses fundamental and methodological issues, such as the crystal structure and signal transduction networks of P2Y receptors, long-term follow-up evidence on the impact of genetic polymorphisms on drug response, and systematic evaluations of long-term antithrombotic strategies. Such work resists translation into public-facing communication narratives in the short term, resulting in relatively limited online traction. Over a longer timescale, however, it is continuously cited and expanded upon by subsequent research, forming the deep skeleton of the field’s knowledge system. Therefore, the “academic-societal attention misalignment” observed here primarily reflects differences in evaluation dimensions and temporal scales: AAS is more sensitive to recent, clinically relevant research that is easily disseminated, whereas traditional citations accumulate persistently around studies with enduring conceptual and methodological contributions. In assessing the impact of P2Y receptor-related cardiovascular research, juxtaposing Altmetric metrics with citation-based metrics—rather than substituting one for the other—serves a dual purpose. This approach helps identify which studies are currently generating spillover effects on clinical practice and policy, while also suggesting that researchers should consider designing differentiated communication strategies for different audiences. Only by fully accounting for structural factors such as publication age, journal influence, and social media biases can the relationship between the Altmetric Attention Score and citation metrics be accurately understood and effectively utilized to guide future research and knowledge translation in the P2Y field.

### 4.7 Limitations

This study has several inherent limitations. First, our analysis was restricted to English-language publications indexed in major bibliographic databases. This selection criterion introduces a coverage bias, as mainstream indexing systems prioritize internationally oriented journals. Consequently, significant research outputs—particularly those focusing on community-based interventions and patient-centered outcomes—may be systematically overlooked.This exclusion risks marginalizing unique clinical evidence rooted in specific regional practices, which could lead to a biased assessment of the field’s structural evolution (Ng et al., 2025). Second, data for the most recent period (2025) are incomplete due to database indexing delays and the “citation lag” phenomenon, so the latest emerging trends may be underrepresented. Third, the descriptive bibliometric indicators used here, such as citation counts and burst strength, map research activity and structural evolution but are proxies for impact rather than direct measures of scientific quality or clinical validity. To address these constraints, future work should integrate semantic modeling and advanced network-based analyses to move beyond keyword co-occurrence, capture contextual nuances in scientific content, and more accurately trace knowledge dissemination across linguistic and disciplinary boundaries.

To address these limitations, future research could adopt complementary methodological strategies. Integrating semantic modeling techniques, such as natural language processing, with advanced network-based analyses would enable the capture of contextual and conceptual nuances in scientific content, moving beyond simple keyword co-occurrence. This approach would facilitate a more accurate tracing of knowledge dissemination across linguistic and disciplinary boundaries. Alternatively, future studies might employ the knowledge triangulation framework proposed by Kokol et al. (2018), which combines quantitative bibliometric mapping with qualitative synthesis. By integrating multiple sources of evidence, this framework provides a structured means to identify emerging research themes and latent knowledge structures that purely quantitative metrics could overlook.

## 5 Conclusion

This study advances beyond traditional bibliometric description by providing a qualitative synthesis of P2Y receptor research in cardiovascular diseases. Integrating bibliometric mapping with qualitative content analysis confirms a fundamental structural transformation in the field, from an initial phase of pharmacological exploration to the current era of precision management. The analysis indicates that future progress will depend not on discovering more potent anticoagulants, but on optimizing de-escalation strategies and leveraging the pleiotropic effects of P2Y receptors in immunothrombosis. These findings provide clinicians and policymakers with data-driven insights for prioritizing resource allocation toward personalized interventions and novel drug delivery systems.

## Data Availability

The raw data supporting the conclusion of this article will be made available by the authors, without undue reservation.
