# Supplementary figures and images for: Knowledge domain and evolutionary trends of P2Y receptors in cardiovascular diseases: a bibliometric and altmetric analysis
Source: Front Pharmacol. 2026 Jan 20;16:1731397. doi: 10.3389/fphar.2025.1731397 (PMC12864444; doi:10.3389/fphar.2025.1731397)

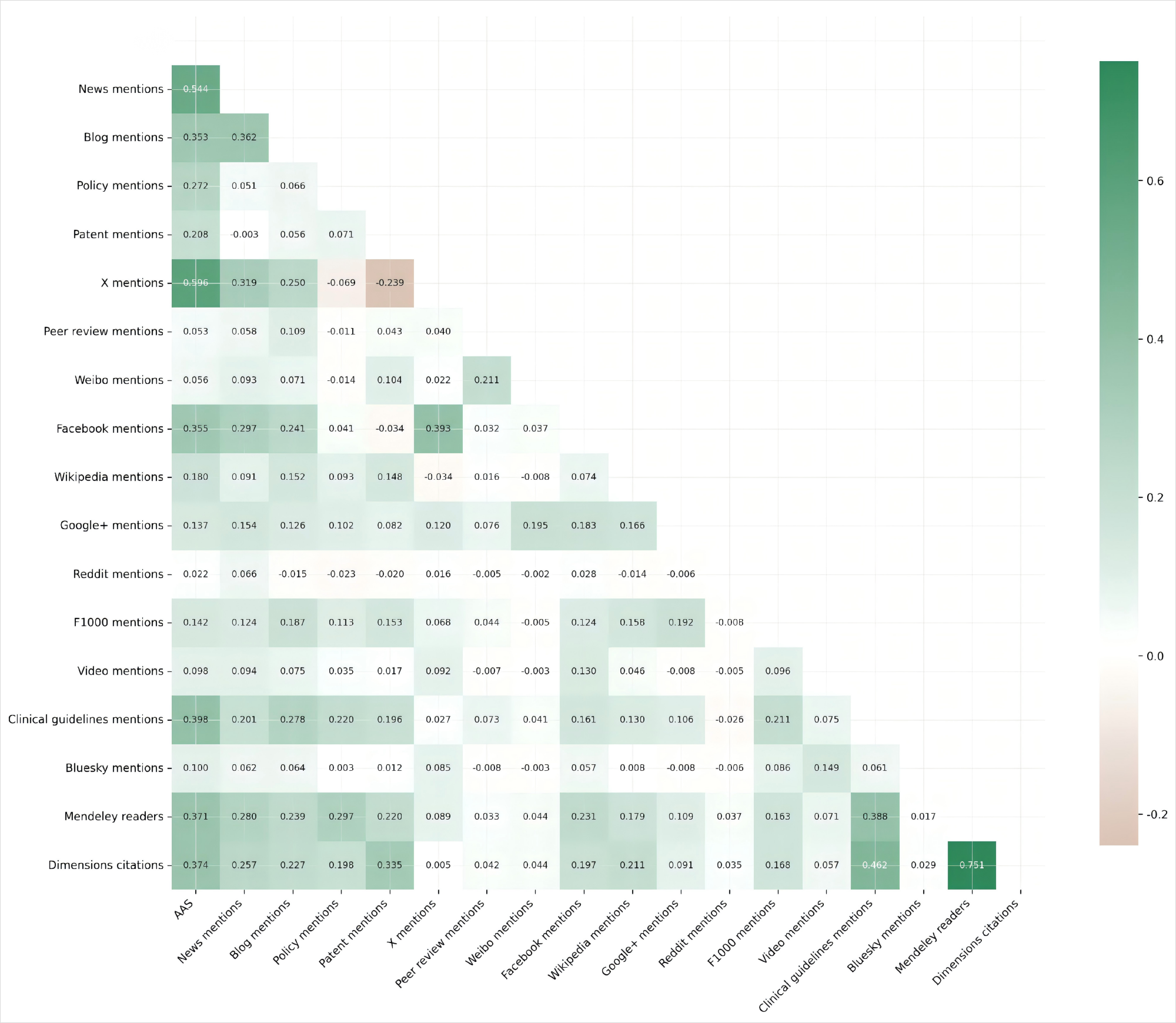

Supplement: Supplementary file 1 [file Image14.png]

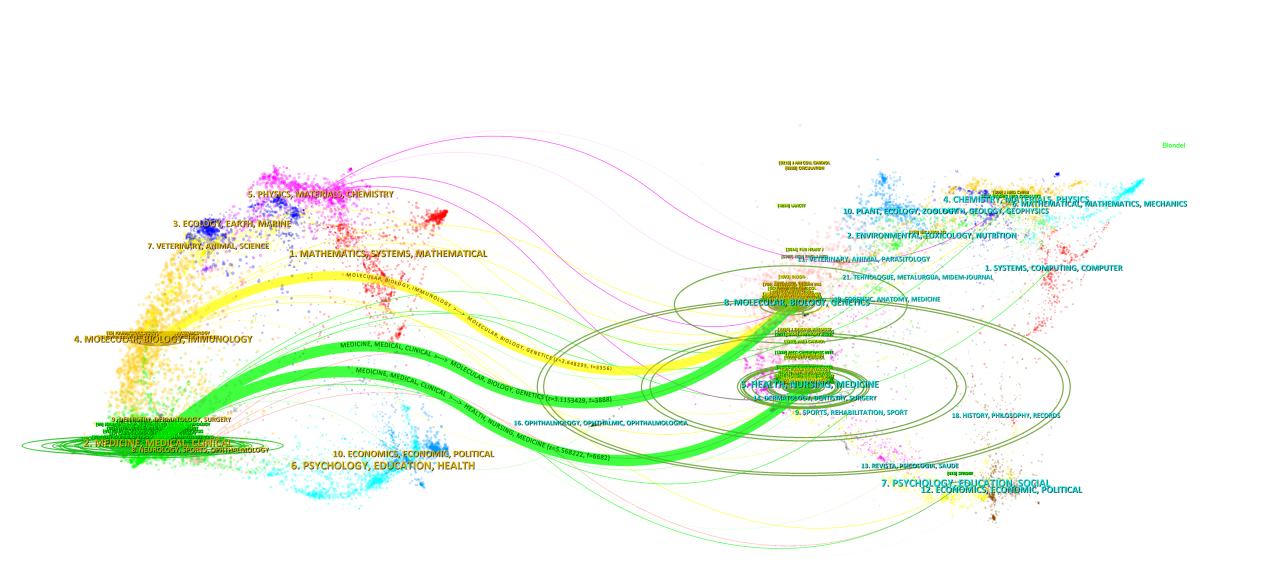

Supplement: Supplementary file 3 [file Image6.tif]

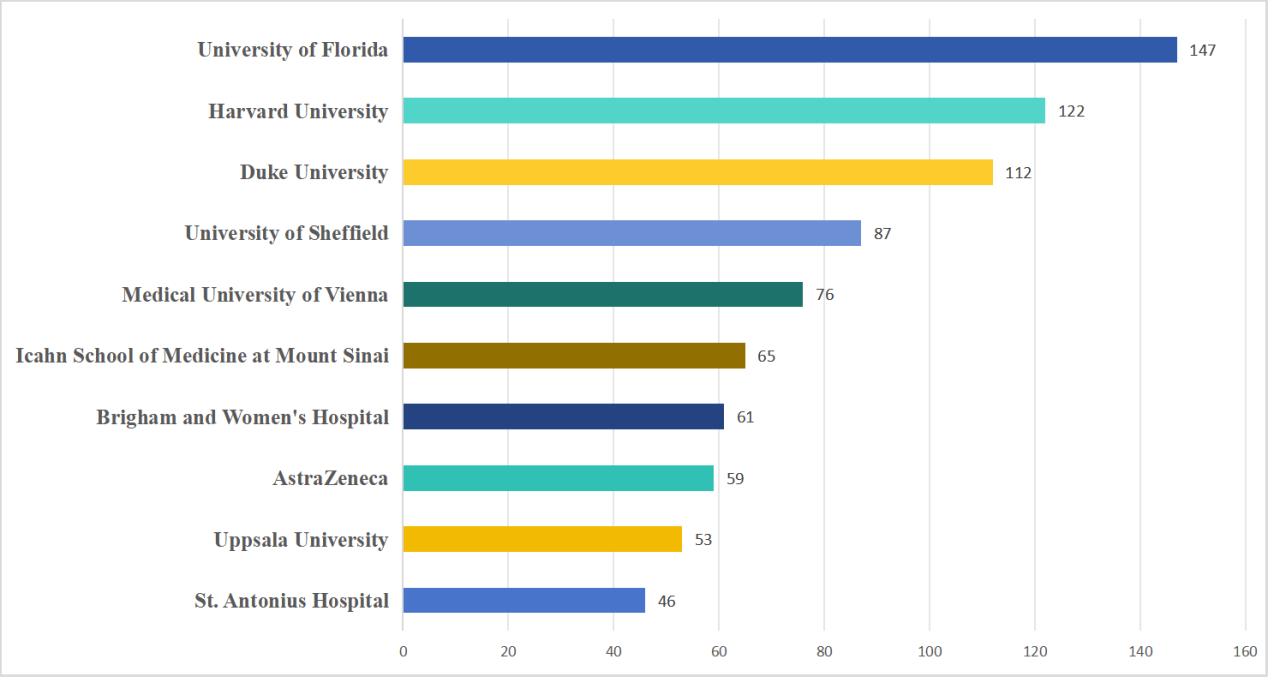

Supplement: Supplementary file 5 [file Image3.tif]

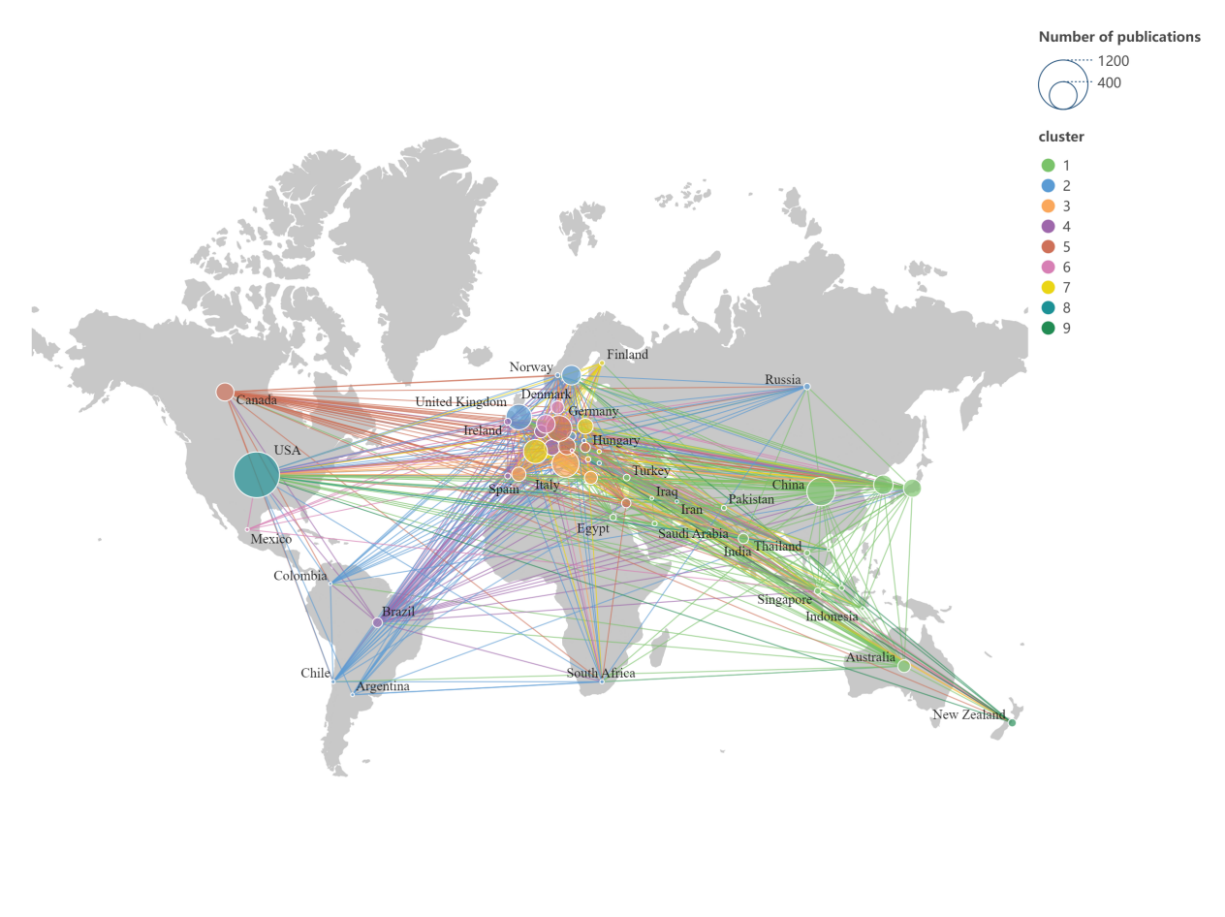

Supplement: Supplementary file 6 [file Image4.tif]

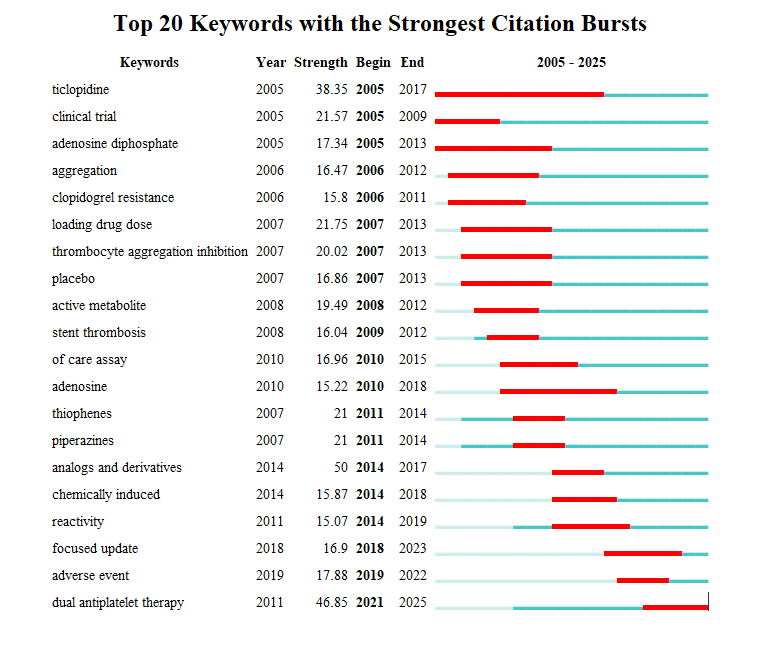

Supplement: Supplementary file 8 [file Image9.tif]

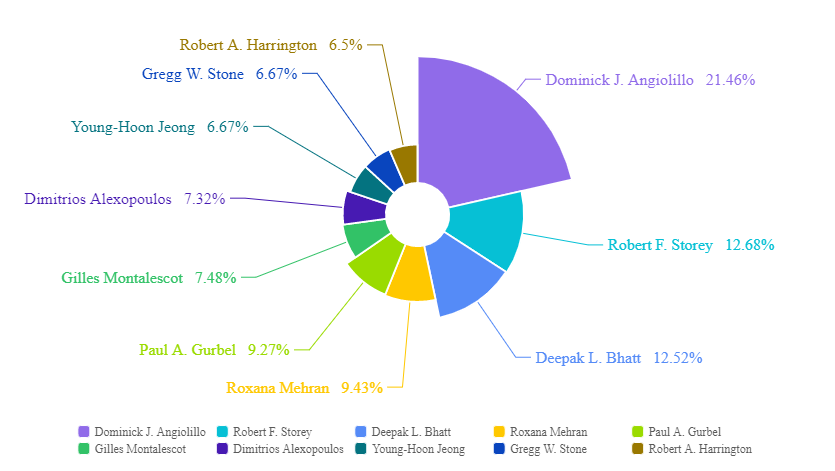

Supplement: Supplementary file 9 [file Image2.tif]

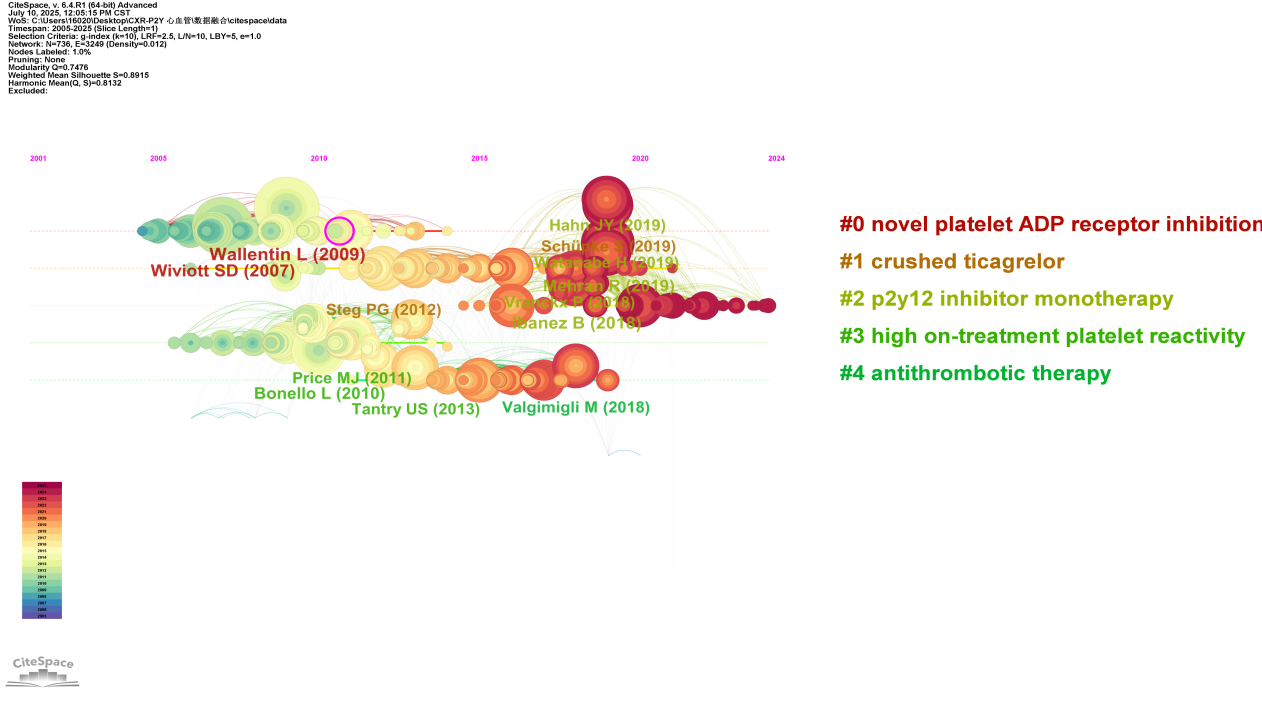

Supplement: Supplementary file 10 [file Image13.tif]

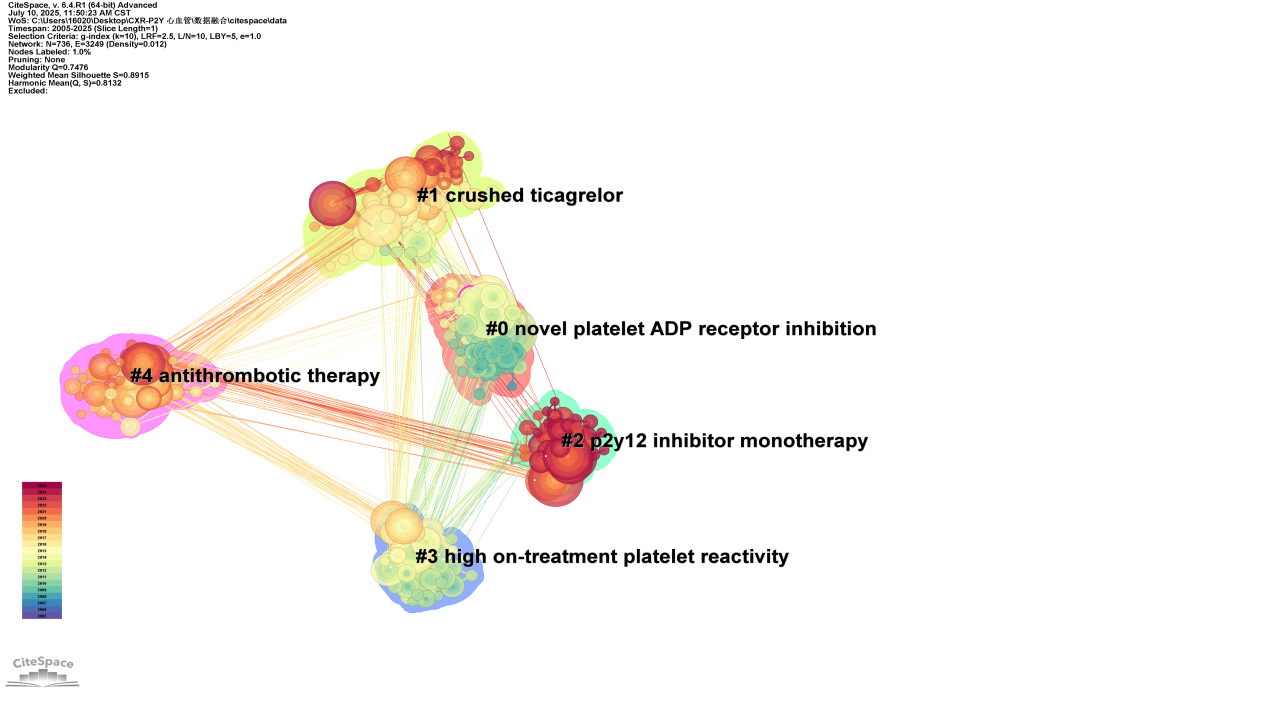

Supplement: Supplementary file 11 [file Image11.tif]

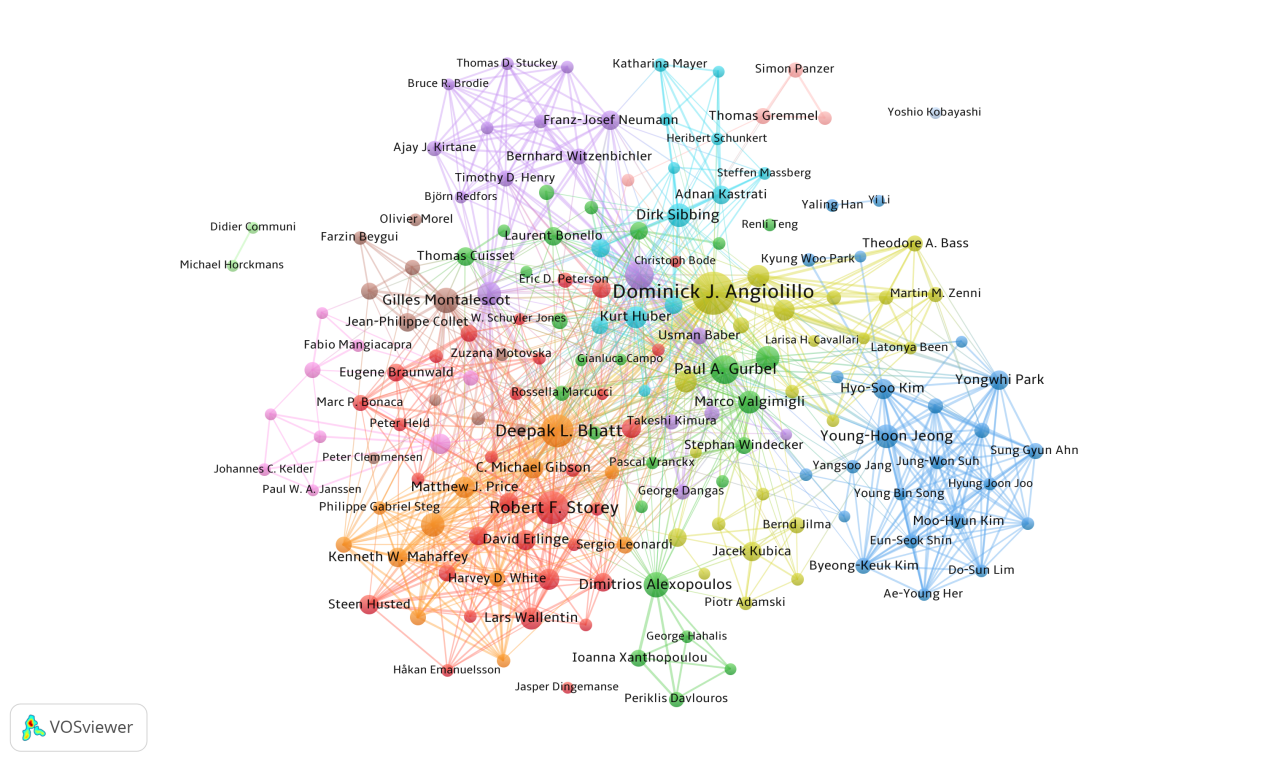

Supplement: Supplementary file 12 [file Image1.tif]

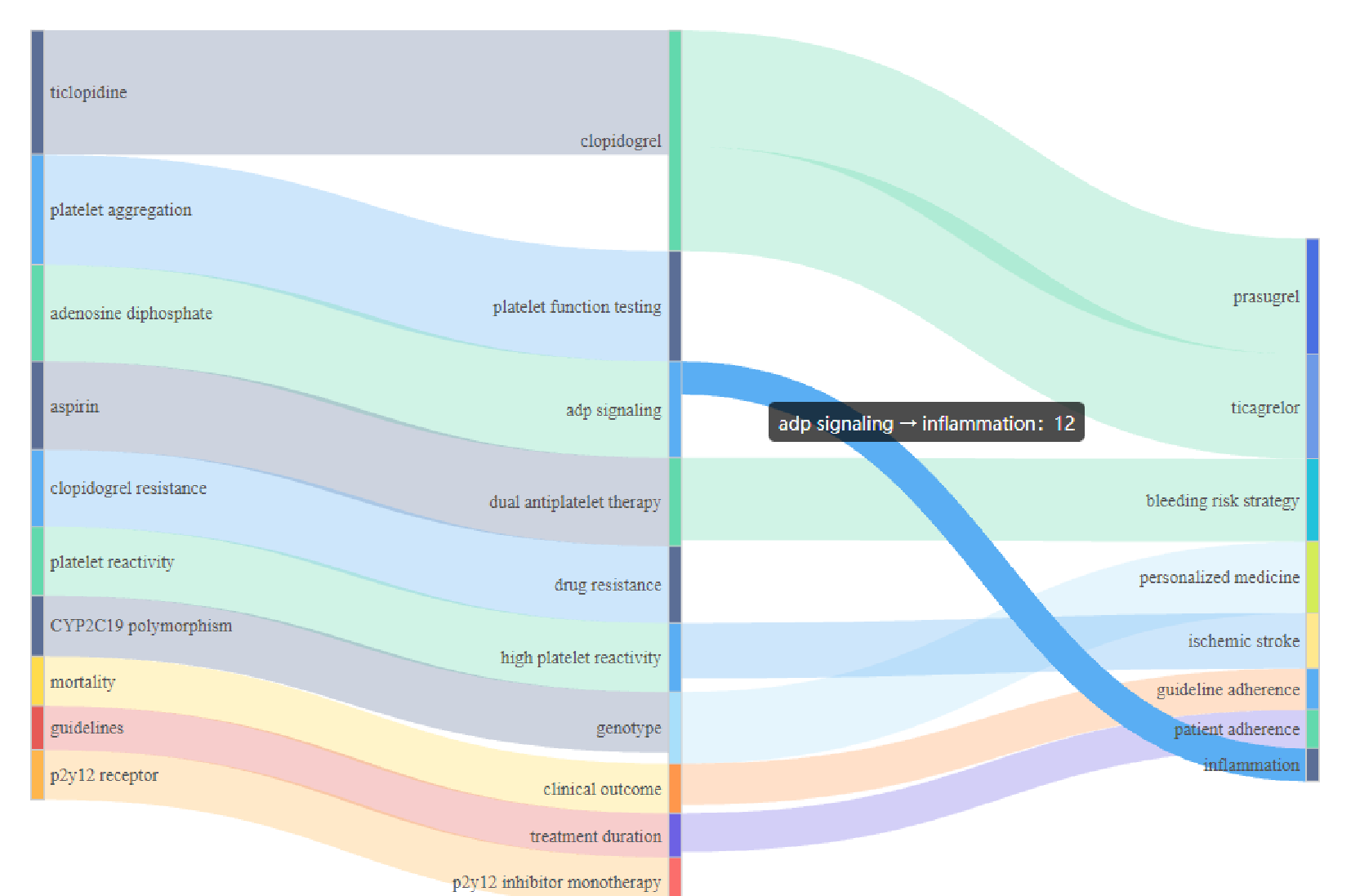

Supplement: Supplementary file 13 [file Image10.tif]

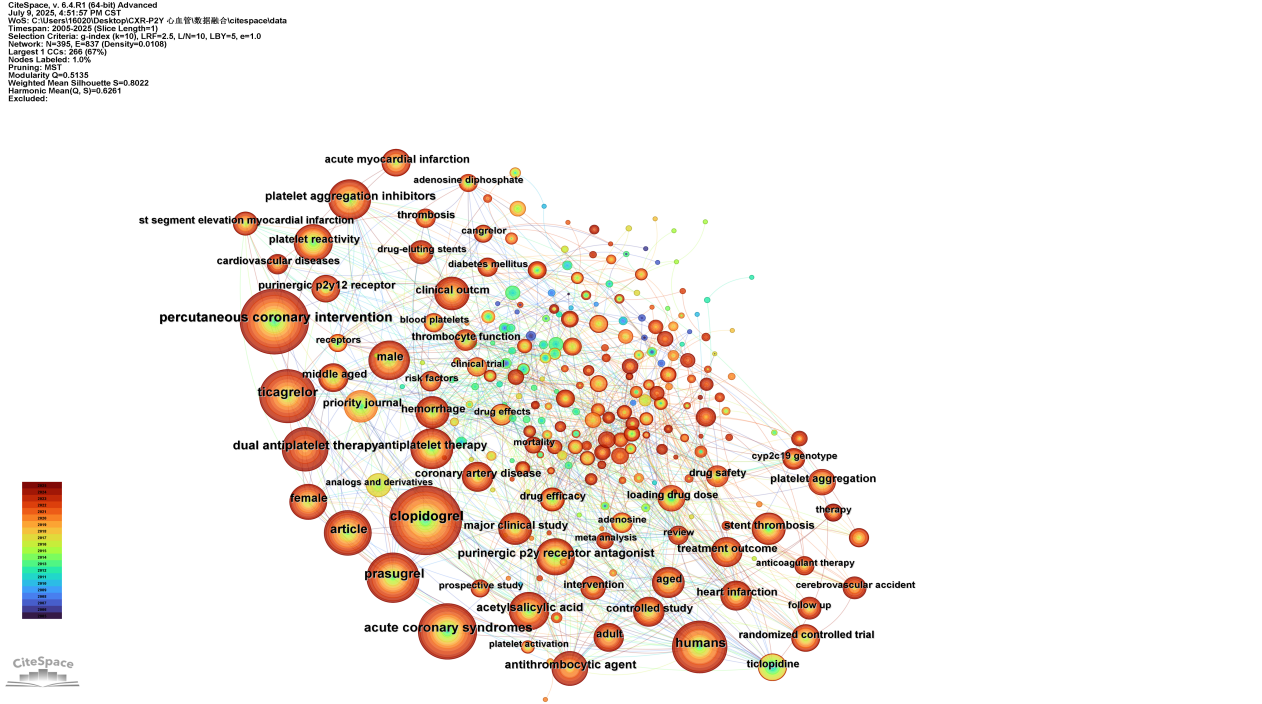

Supplement: Supplementary file 14 [file Image7.tif]

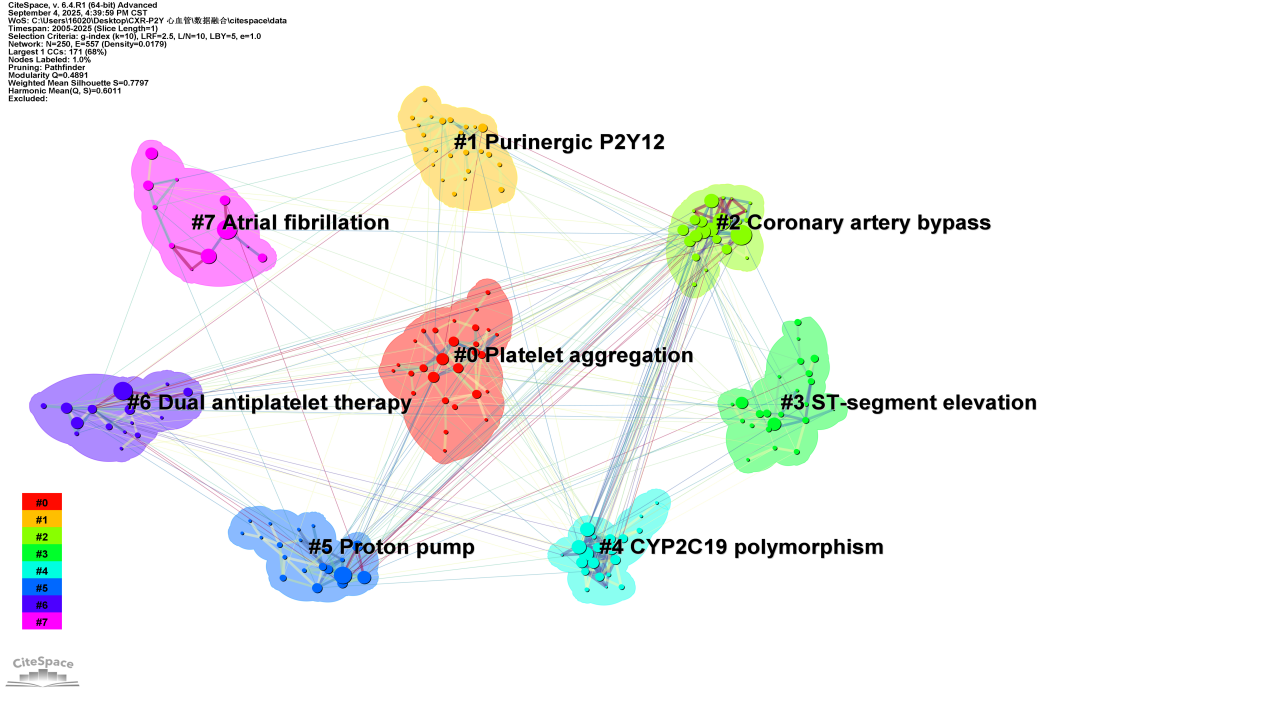

Supplement: Supplementary file 16 [file Image8.tif]

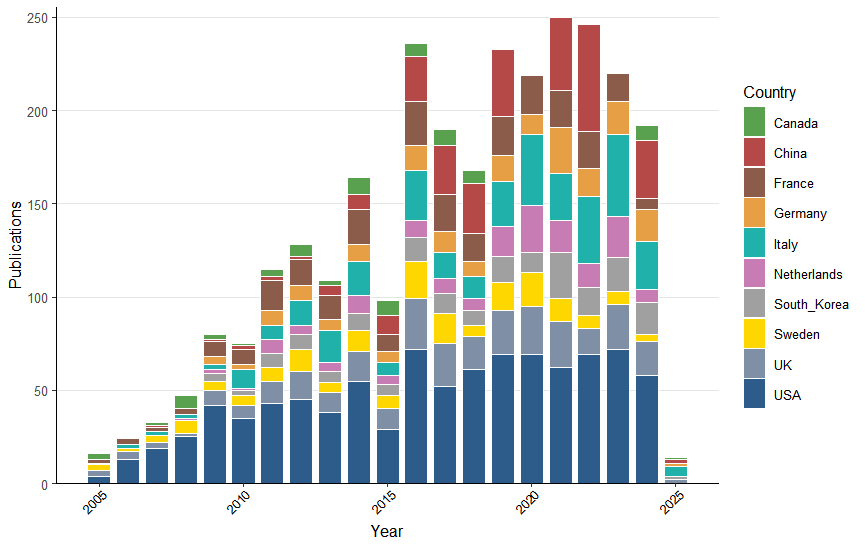

Supplement: Supplementary file 17 [file Image5.tif]

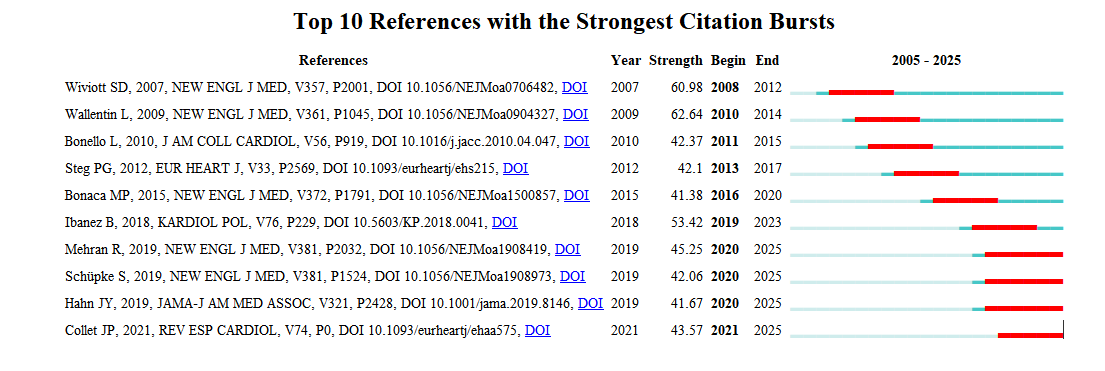

Supplement: Supplementary file 19 [file Image12.tif]
