# Supplementary material for: Knowledge domain and evolutionary trends of P2Y receptors in cardiovascular diseases: a bibliometric and altmetric analysis
Source: Front Pharmacol. 2026 Jan 20;16:1731397. doi: 10.3389/fphar.2025.1731397 (PMC12864444; doi:10.3389/fphar.2025.1731397)
Supplement: Supplementary file 2 [file Table4.pdf]

TABLE A4 P2Y in highly co-cited journals in the field of CVDS

Rank	Co-cited journals	Co-Citations	IF(2023)	JCR division
1	Journal of the American College of Cardiology	8352	21.7	Q1
2	Circulation	8202	35.6	Q1
3	The New England Journal of Medicine	7381	96.3	Q1
4	European Heart Journal	4947	38.1	Q1
5	The Lancet	3886	98.4	Q1
6	Thromb Haemostasis	2806	5	Q1
7	Journal of the American Medical Association	2459	63.5	Q1
8	Journal of Thrombosis and Haemostasis	2300	5.5	Q1
9	American Heart Journal	2030	3.7	Q1
10	American Journal of Cardiology	1808	2.3	Q2
