# Supplementary material for: Knowledge domain and evolutionary trends of P2Y receptors in cardiovascular diseases: a bibliometric and altmetric analysis
Source: Front Pharmacol. 2026 Jan 20;16:1731397. doi: 10.3389/fphar.2025.1731397 (PMC12864444; doi:10.3389/fphar.2025.1731397)
Supplement: Supplementary file 4 [file Table1.pdf]

TABLE A1 The top 10 most productive institutions and countries in P2Y cardiovascular research.

Rank	Insititution	TP	Rank	Author	TP	H-index
1	University of Florida	147	1	Dominick J. Angiolillo	132	105
2	Duke University	112	2	Robert F. Storey	78	93
3	Harvard University	122	3	Deepak L. Bhatt	77	180
4	Brigham and Women's Hospital	61	4	Roxana Mehran	58	167
5	University of Sheffield	87	5	Paul A. Gurbel	57	77
6	Icahn School of Medicine at Mount Sinai	65	6	Gilles Montalescot	46	118
7	Sungkyunkwan University	37	7	Dimitrios Alexopoulos	45	52
8	AstraZeneca	59	8	Young-Hoon Jeong	41	34
9	Columbia University	32	9	Gregg W. Stone	41	182
10	Uppsala University	53	10	Robert A. Harrington	40	135
