# Supplementary material for: Knowledge domain and evolutionary trends of P2Y receptors in cardiovascular diseases: a bibliometric and altmetric analysis
Source: Front Pharmacol. 2026 Jan 20;16:1731397. doi: 10.3389/fphar.2025.1731397 (PMC12864444; doi:10.3389/fphar.2025.1731397)
Supplement: Supplementary file 7 [file Table2.pdf]

TABLE A2 The top 10 countries contributing to publications in P2Y cardiovascular research.

Rank	Country	TLS	TP	Percentage (%)
1	USA	1416	941	20.27%
2	China	318	367	7.90%
3	Italy	892	333	7.17%
4	Germany	946	298	6.42%
5	United Kingdom	1074	292	6.29%
6	France	873	252	5.43%
7	South Korea	256	177	3.81%
8	Sweden	562	172	3.70%
9	Netherlands	660	161	3.47%
10	Canada	552	150	3.23%
