# Supplementary material for: Knowledge domain and evolutionary trends of P2Y receptors in cardiovascular diseases: a bibliometric and altmetric analysis
Source: Front Pharmacol. 2026 Jan 20;16:1731397. doi: 10.3389/fphar.2025.1731397 (PMC12864444; doi:10.3389/fphar.2025.1731397)
Supplement: Supplementary file 15 [file Table3.pdf]

TABLE A3: High-yield journals of P2Y in the field of CVDS

Rank	Journal	TP	IF(2023)	JCR division
1	Thrombosis and Haemostasis	72	5	Q1
2	Journal of the American College of Cardiology	70	21.7	Q1
3	Platelets	66	2.5	Q3
4	JACC: Cardiovascular Interventions	52	11.7	Q1
5	American Heart Journal	40	3.7	Q1
6	Circulation	40	35.6	Q1
7	Journal of the American Heart Association	38	63.5	Q1
8	American Journal of Cardiology	36	2.3	Q2
9	European Heart Journal	34	38.1	Q1
10	Journal of Thrombosis and Thrombolysis	34	5.5	Q1
